# Supplementary material for: The Epstein-Barr virus deubiquitinating enzyme BPLF1 regulates the activity of topoisomerase II during productive infection
Source: PLoS Pathog. 2021 Sep 20;17(9):e1009954. doi: 10.1371/journal.ppat.1009954 (PMC8483405; doi:10.1371/journal.ppat.1009954)
Supplement: S1 Table — (DOCX) [file ppat.1009954.s001.docx]

| **Table S1. Reagents used in this paper** |  |  |
| --- | --- | --- |
| **Reagent** | **Source** | **Identifier** |
| **Antibodies** | | |
| Mouse monoclonal Anti-β-Actin clone AC-15, | Sigma-Aldrich | Cat# A5441, RRID:AB_476744 |
| Mouse monoclonal anti-FLAG | Sigma-Aldrich | Cat# F3165, RRID:AB_259529) |
| Rabbit polyclonal anti-TOP1 | Bethyl Laboratories | Cat# A302-589A, RRID:AB_2034865 |
| Rabbit polyclonal anti-TOP2α | Bethyl Laboratories | Cat# A300-054A, RRID:AB_221392 |
| Rabbit polyclonal anti-TOP2β | Bethyl Laboratories | Cat# A300-950A, RRID:AB_805860 |
| Rabbit polyclonal anti-53BP1 | Bethyl Laboratories | Cat# A300-272A, RRID:AB_185520 |
| Phospho-Histone H2A.X (Ser139) (20E3) Rabbit mAb antibody | Cell Signaling Technology | Cat# 9718, RRID:AB_2118009 |
| Mouse monoclonal anti-Ub (P4D1) | Santa Cruz Biotechnology | Cat# sc-8017 AC, RRID:AB_2762364 |
| Mouse monoclonal anti-EBV ZEBRA (BZ1) | Santa Cruz Biotechnology | Cat# sc-53904, RRID:AB_783257 |
| Mouse Monoclonal anti-Human BRCA1 (D-9) | Santa Cruz Biotechnology | Cat# sc-6954, RRID:AB_626761 |
| Mouse Monoclonal anti-Sumo 2+3 antibody [8A2] | Abcam | Cat# ab81371, RRID:AB_1658424 |
| Rat monoclonal anti-EBV-BPLF1 | MAB core facility, Helmholtz Center, Munich, Germany  [1] | N/A |
| Mouse monoclonal anti-EBV-BMRF1 | Dr. Jaap M. Middeldorp (VU University Medical Center,Amsterdam, NL) | N/A |
| Rat polyclonal anti-EBV-BFRF3 | Dr. Jaap M. Middeldorp (VU University Medical Center,Amsterdam, NL) | N/A |
| Donkey anti-Mouse IgG (H+L) Highly Cross-Adsorbed Secondary Antibody, Alexa Fluor 555 | Thermo Fisher Scientific | Cat# A-31570, RRID:AB_2536180 |
| Donkey Anti-Rabbit IgG (H+L) Antibody, Alexa Fluor 488 Conjugated | Thermo Fisher Scientific | Cat# A-21206, RRID:AB_2535792 |
| **Bacterial and virus strains** | | |
| rB95-8/p2089, recombinant wild type EBV B95.8 strain | [2] | N/A |
| B769, recombinant BPLF1^C61A^ mutant EBV | [3] | N/A |
| **Biological samples** |  |  |
| Buffy coats | Karolinska University Hospital, Stockholm, SE | N/A |
| **Chemicals, peptides, and recombinant proteins** | | |
| IGEPAL CA-630 | Sigma-Aldrich | I3021; CAS: 9002-93-1 |
| Sodium dodecyl sulphate | Sigma-Aldrich | L3771;CAS:151-21-3 |
| N-Ethylmaleimide | Sigma-Aldrich | E1271; CAS:128-53-0 |
| Iodoacetamide | Sigma-Aldrich | I1149; CAS:144-48-9 |
| Sodium deoxycholate monohydrate | Sigma-Aldrich | D5670;CAS:145224-92-6 |
| Triton X-100 | Sigma-Aldrich | T9284; CAS:9002-93-1 |
| Bovine serum albumin | Sigma-Aldrich | A7906; CAS:9048-46-8 |
| Tween-20 | Sigma-Aldrich | P9416; CAS: 9005-64-5 |
| Trizma base | Sigma-Aldrich | 93349; CAS:77-86-1 |
| Ethylenediaminetetraacetic acid disodium salt dehydrate | Sigma-Aldrich | E4884; CAS:6381-92-6 |
| Doxycycline cyclate | Sigma-Aldrich | D9891; CAS: 24390-14-5 |
| MG132 | Sigma-Aldrich | M7449; EC: 200-664-3 |
| Etoposide | Sigma-Aldrich | E1383; CAS:33419-42-0 |
| Neocarzinostain | Sigma-Aldrich | N9162; CAS: 9014-02-2 |
| Imidazole | Sigma-Aldrich | I5513; CAS: 288-32-4 |
| Methylthiazolyldiphenyl-tetrazolium bromide | Sigma-Aldrich | M5655; CAS:298-93-1 |
| Polybrene | Sigma-Aldrich | TR-1003-G; |
| RNase A | Invitrogen | Cat# 12-091-021 |
| DNAzol | Invitrogen | Cat# 10503-027 |
| Micrococcal nuclease | Thermo Fisher Scientific | Cat# 88216 |
| Complete protease inhibitors cocktail tablets | Roche Diagnostic | Cat#04693116001 |
| Phosphatase inhibitor cocktail | Roche Diagnostic | Cat#04906837001 |
| Camptothecin | Selleckchem | Cat#S1288 |
| **Experimental models: Cell lines** | | |
| HeLa | ATCC | RR-B51S |
| HEK293T | ATCC | CRL3216 |
| HEK-rtTA-BPLF1 | This paper | N/A |
| HEK-rtTA-BPLF1^C61A^ | This paper | N/A |
| HEK293-EBV-BPLF1  HEK293-EBV-BPLF1^C61A^ | [3] | N/A |
| LCL-BPLF1 (doxycycline-inducible BZLF1) | This paper | N/A |
| LCL-BPLF1^C61A^ (doxycycline-inducible BZLF1) | This paper | N/A |
| TK6  TK6-TDP2^-/-^ | Hoa et al, Mol Cell 2016 |  |
| TK6 (doxycycline-inducible BZLF1) | This paper | N/A |
| TK6-TDP2^-/-^ (doxycycline-inducible BZLF1) | This paper | N/A |
| **Oligonucleotides** | | |
| For qPCR primers see Table S1 |  |  |
| Cloning of BZLF1 to pTRIPZ Vector, Forward 5´- CGACCGGTATGATGGACCCAAACTCGAC-3´; Reverse 5´- CGACGCGTTTAGAAATTTAAGA GA TCCTCGTGT-3´ | This paper | N/A |
| **Recombinant DNA** | | |
| pCW57.1 | gift from David Root | Addgene #41393 |
| FLAG-TOP2α | This paper | N/A |
| FLAG-TOP2α-ΔCTD | This paper | N/A |
| His-BPLF1 | Gupta, S., et al, PLoS Pathogens 2019 | N/A |
| 12URA-B | gift from Scott Gradia | Addgene #48304 |
| pTRIPZ lentiviral vector | Dharmacon; ThermoScientific | Cat#RHS4750 |
| psPAX2 | gift from Didier Trono | Addgene # 12260 |
| pMD2.G | gift from Didier Trono | Addgene # 12259 |

References

[1] M. van Gent, S.G. Braem, A. de Jong, N. Delagic, J.G. Peeters, I.G. Boer, P.N. Moynagh, E. Kremmer, E.J. Wiertz, H. Ovaa, B.D. Griffin, M.E. Ressing, Epstein-Barr virus large tegument protein BPLF1 contributes to innate immune evasion through interference with toll-like receptor signaling, PLoS Pathog 10(2) (2014) e1003960

[2] H.J. Delecluse, T. Hilsendegen, D. Pich, R. Zeidler, W. Hammerschmidt, Propagation and recovery of intact, infectious Epstein-Barr virus from prokaryotic to human cells, Proc Natl Acad Sci U S A 95(14) (1998) 8245-50

[3] S. Gupta, P. Yla-Anttila, S. Callegari, M.H. Tsai, H.J. Delecluse, M.G. Masucci, Herpesvirus deconjugases inhibit the IFN response by promoting TRIM25 autoubiquitination and functional inactivation of the RIG-I signalosome, PLoS Pathog 14(1) (2018) e1006852
