# Supplementary material for: The Epstein-Barr virus deubiquitinating enzyme BPLF1 regulates the activity of topoisomerase II during productive infection
Source: PLoS Pathog. 2021 Sep 20;17(9):e1009954. doi: 10.1371/journal.ppat.1009954 (PMC8483405; doi:10.1371/journal.ppat.1009954)
Supplement: S2 Table — (DOCX) [file ppat.1009954.s002.docx]

| **Table S2: qPCR primers used in this paper** | | |
| --- | --- | --- |
| **Gene** | **Forward primer(5´-3´)** | **Reverse primer(5´-3´)** |
| TOP1 | AGTGGAAAGAAGTCCGGCATGA | GCCAGTCCTTCTCACCCT TGAT |
| TOP2α | AAGCCCAGCAAAAGGTTCCA | TGGCTTCAACAGCCTCCAAT |
| TOP2β | GGTTCGTGTAGAGGGGTCAA | CCCAGTTTCATCCAATTTGTC |
| BPLF1 | CATACACCGTGCGAAAAGAA | GATGGCGGGTAATACATGCT |
| MLN51 | CAAGGAAGGTCGTGCTGGTT | ACCAGACCGGCCACCAT |
| EBNA1 | GGCAGTGGACCTCAAAGAAG | CTATGTCTTGGCCCTGATCC |
| EF1α | CTGAACCATCCAGGCCAAAT | GCCGTGTGGCAATCCAAT |
