# Supplementary material for: The Epstein-Barr virus deubiquitinating enzyme BPLF1 regulates the activity of topoisomerase II during productive infection
Source: PLoS Pathog. 2021 Sep 20;17(9):e1009954. doi: 10.1371/journal.ppat.1009954 (PMC8483405; doi:10.1371/journal.ppat.1009954)
Supplement: Attachment — Submitted filename: Rebuttal letter.docx [file ppat.1009954.s010.docx]

Re:PPATHOGENS-S-21-01403-R1

Dear Editors,

Please find enclosed a revised version of the above manuscript. We are pleased by the overall positive assessment of the manuscript and would like to thank the reviewers for many insightful comments that made us realize that some of the findings were not adequately described and discussed. We have carefully considered all the comments and modified the manuscript accordingly or, when relevant, explained our reasons for not doing so.

In particular:

1. Experiment on the effect of TDP2 knockdown on the induction of DNA damage in productively infected cells suggested by reviewer #1 were not informative due to the high levels of endogenous DNA damage in TDP2-/- cells.

2. We are puzzled by the comment of reviewer #2 requesting that the effect of BPLF1 be tested in cells supporting the full lytic cycle. The key features of the phenotype observed in cells expressing inducible BPLF1/BPLF1^C61A^ were reproduced upon induction of the productive cycle in three sets of EBV positive cell lines: HEK293-EBV-BPF1/BPLF1^C61A^, LCL-BPLF1/BPLF1^C61A^, and TK6/TK6-TDP2^-/-^. As documented in the supplementary figures all cell lines express late antigens upon induction and we assume therefore that they are capable to complete the full lytic cycle. We have interpreted the comment of the reviewer as a request for analysis of virus release. When a meaningful conclusion could be drawn, we have added this information and discussed the significance of our findings. The reviewer also requests confirmation of direct interaction between BPLF1 and TOP2. This evidence is provided in Fig. 1D (reciprocal co-immunoprecipitation) and Fig. S2 (pull-down assays using recombinant proteins). We are commenting on the possible reasons for the different efficiency of interaction in IP versus PD, which suggests that additional factors (post-translational modification and/or additional binding partners) may strengthen the interaction in cells. We have modified the text to clarify this issue. As stated in the text we are unable to perform co-IP of endogenous TOP2 and BPLF1 in induced cells because the available BPLF1 antibodies do not detect the endogenous protein. Despite this technical shortcoming, we feel that the capacity of BPLF1 to interact with TOP2 and regulate its activity is amply demonstrated by the entire set of experiments presented in the paper.

3. we have clarified the issue of TOP2 SUMOylation risen by reviewer #3.

A point-by-point response to the specific comments of the three reviewers is included below. We wish to thank you for a careful and useful assessment of the paper and hope that you will find the revised version suitable for publication in PLoS Pathogens.

Sincerely yours

Maria Masucci

**Point-by-point response to reviewers - PPATHOGENS-S-21-01403**

**Reviewer #1**

We thank the reviewer for the positive assessment of our work.

**Specific comments:**

1. We have commented on the contribution of BPLF1 to virus replication and added four relevant references in the Discussion (pages 19 to 21). We have performed the analysis of DNA damage in induced TK6-TDP2^-/-^ cells, but the experiments were not informative due to the high endogenous levels of γH2AX in this cell line. Representative micrographs illustrating the expression of γH2AX in untreated and Etoposide treated TK6 and TK6-TDP2^-/-^ is included at the end of this rebuttal letter for inspection by the reviewer (Figure 1). To compensate for this technical limitation, we have added new data on the induction of DNA damage in the LCL-BPLF1/BPLF1^C61A^ pair (see new Fig. 6G and 6H, related figure legends and Results page 15). Although not requested by the reviewer, the data further support the involvement of the DUB activity in the regulation of DNA damage during productive infection.

**Minor issues:**

1. the reviewer correctly points out that BPLF1 overexpression is associated with a broad deubiquitination of cellular proteins, which casts some doubt on the specificity of the effect. We fully appreciate that the strong deubiquitinase activity of BPLF1 is an issue and that certain substrates may be deubiquitinated only when BPLF1 is overexpressed. However, the deubiquitination of TOP2 is not an artifact of overexpression because: a) it is observed in cells where TOP2 ubiquitination is induced by treatment with Etoposide and the level of global ubiquitination is more affected in the treated cells (compare Fig. 2A lower panels) confirming that what we are measuring is an Etoposide-induced effect; b) not every protein is deubiquitinated in BPLF1 transfected cells, as confirmed by the failure to rescue the proteasomal degradation of TOP1 induced by Cpt treatment. Furthermore, as the reviewer also points out, deubiquitination of TOP2 is observed in induced cells where BPLF1 is expressed at physiological levels and the overall ubiquitination is not significantly affected. We have clarified this issue in the text (see Results page 9).
2. We feel that the reviewer may have partially misunderstood the experiment. Fig.S3 shows that BPLF1 stabilizes TOP2cc induced by Etoposide. This conclusion is based on a comparison of the levels to TOP2cc clearance (decreased intensity of the high mw smear from during treatment for 6h) in Dox- (BPLF1 negative) versus Dox+ (BPLF1 positive cells). While a significant reduction of TOP2ccs is observed in the Dox- cells, the amount of TOP2ccs remains stable in BPLF1 expressing cells. Similar stabilization of TOP2ccs is observed in MG132 treated cells independently of Dox treatment confirming that the clearance is mediated by proteasomal degradation. The reviewer is correct in noticing minor differences in the intensity of the TOP2ccs in Fig. 2B 0.5h treatment +/- Dox and revised Fig. S4A 1h treatment +/- Dox. These are however small and not reproducible between experiments. We are commenting on this issue in the text (see Results page 10). It is important to stress that the effect of Etoposide on the induction of TOP2ccs is extremely rapid (observed within minutes) and rapidly reversible upon removal of the drug, which requires great care during washing and handling of the treated cells. In our preliminary experiments (not extensively described in the manuscript due to space limitation) we have compared the levels of TOP2ccs after continuous treatment with a range of Etoposide concentrations for various lengths of time and found no significant difference during the first 30 min in BPLF1 positive and negative cells. We are therefore confident that the differences observed at 6h cannot be ascribed to impaired formation of TOP2ccs. A dot blot illustrating the comparable levels of TOP2ccs in cells treated for 15 min with various amounts of Etoposide is now included in Supporting Information, new Fig S3.
3. The reviewer is correct in stating that several publications have reported that the deubiquitinase activity of the herpesvirus large tegument proteins are not essential for virus replication. This is confirmed in our paper by the production of infectious virus and the establishment of LCLs carrying the BPLF1-C61A mutant. However, we and others have shown that the activity is required for efficient virus replication and the regulation of other aspects of the virus life cycle (see Ref. 30, new Ref. 58-62 and Figure 2 attached to this rebuttal letter). We have commented on this aspect of our findings in the Discussion (page 21).

**Reviewer #2**

We thank the reviewer for several insightful comments that have revealed our failure to clearly describe and discuss some of the data.

**Specific comments**

1. The reviewer laments the lack of validation of our finding in a virus replication model. We would like to emphasize that wherever possible the key findings obtained with BPLF1 inducible cells (Fig. 1,2,3,4) were validated in EBV carrying cells where the productive cycle was induced by expression of a Dox-regulated BZLF1 (Fig. 5 using HEK293-EBV-BPLF1/BPLF1^C61A^, Fig.6 using LCL-BPLF1/BPLF1^C61A^ and new Fig. 7 using the LCL pair TK6/TK6-TDP2^-/-^). Thus, the HEK293 model suggested by the reviewer is already included in the paper along with LCLs produced with either the EBV BAC expressing catalytically active or inactive BPLF1 or by spontaneous outgrowth from a mononucleosis patient. In the induced EBV positive cells, we have confirmed the expression of late viral proteins which is a bona fide correlate of completion of the lytic cycle (revised Fig S6 and S7). The reviewer suggests that the release of infectious virus is the only reliable measure of completed virus replication. This is of course correct, and we have included data confirming that cells lacking TDP2 produce less infectious virus (revised Fig. 7B and related text in Results page 16), which supports the involvement of the BPLF1/TOP2/TDP2 pathways in the regulation of virus production. We have investigated the production of infectious virus in the HEK293-EBV-BPLF1/BPLF1^C61A^ and LCL-BPLF1/BPLF1^C61A^ cells pairs and found that expression of the mutants is regularly associated with impaired virus production. However, the interpretation of these findings in terms of TOP2 regulation is not straightforward since BPLF1 may affect EBV DNA replication and virus production at several levels including the deNEDDylase dependent inactivation of CRL ligases and stabilization of cellular CDT1 (Gastaldello et al. NCB, 2010), and the inhibition of the IFN response, (Gupta et al. PLoS Pathogens, 2018). Therefore, the data are not included in the manuscript, but a summary of the results is included at the end of this rebuttal letter for inspection by the reviewers. We have not performed TOP2 knockdown experiments partly because the TOP2α knockdown is not viable but mainly because the involvement of TOP2 in EBV replication, as documented in numerous publications, would prevent a meaningful interpretation of the findings.
2. Regarding the interaction of BPLF1 with TOP2 see also response Reviewer #1. We appreciate that our description of the co-immunoprecipitation and pull-down assays was unclear, and we have therefore modified the text (see Results page 8-9). Global ChIP assays are indeed an interesting suggestion but outside of the scope of this paper. We are showing that BPLF1 deubiquitinates TOP2 trapped in cleavable complexes (Figure 4A) which unequivocally places the viral enzyme in a complex containing DNA-bound TOP2.

**Minor issues**

1. Regarding the usage of comma versus dot, we have corrected all the figure as suggested.
2. Regarding the interaction of BPLF1 with TOP2, our co-immunoprecipitation experiments show that interaction occurs in cells, and the pulldown assays performed with recombinant proteins confirm that the interaction is direct. However, based on comparison of the IP and PD results, we are commenting on the possibility that the interaction may be strengthened by factors present in cells, such as posttranslational modifications or additional binding partners. We acknowledge that our description and discussion of this finding may have been confusing, and we have modified the relevant text accordingly (see Results page 8-9).
3. We are surprised by the failure of the reviewer to appreciate the lack of 53BP1 foci in BPLF1 positive Etoposide treated cells in Figure 3A and Figure S4. In Figure 3A where HEK293 cells are used, the Etoposide treated BPLF1 positive cells show only diffuse fluorescence, like untreated cells, while foci are visible in the BPLF1 negative cells found in the same image. Due to the background fluorescence, the number of foci per cell rather than the total fluorescence intensity was quantified. In Figure S4 using HeLa cells, the background 53BP1 fluorescence is significantly lower and only the Etoposide induced foci are detected. In this figure, two BPLF1 positive cells show no foci while foci are detected in BPLF1 negative cells. We have clarified this point in the corresponding figure legend and Results (page 11). To facilitate assessment of the data, the 53BP1 and BRCA1 foci negative BPLF1 positive cells are highlighted in the revised Fig. S4 and large size images are shown in Fig. 3A.
4. Figure 6 was corrected as suggested

**Reviewer #3**

We thank the reviewer for the positive assessment of our work

**Specific comments**

1. We apologize for the poor formulation of this sentence that was corrected as suggested.
2. We acknowledge that our discussion of the role of SUMOylation in the regulation of TOP2cc clearance may have been confusing and we have modified the text to clarify our arguments (see Discussion pages 18-19). As noticed by the reviewer, the published literature suggests that ubiquitination and SUMOylation of TOP2 may occur at different residues since SUMOylation serves as the recognition site for ubiquitination of TOP2 by the RNF4 ligase. However, mixed Ub/UbL chains have been identified in numerous studies and RNF4-dependent ubiquitination of SUMOylated TOP2 on Lys residues in SUMO cannot be excluded. Furthermore, other SUMO-independent Ub ligases may also be involved. The question of which residues are involved in either type of modification is interesting but not within the scope of our paper. In the Discussion, we aim to provide an overview of a very complex and extensive literature that illustrates multiple mechanisms by which SUMOylation may regulate TOP2 stability and activity. Our findings support a model based on published data showing that SUMOylation of DNA-trapped TOP2 by the ZNF451 ligase is required for the TDP2-dependent non-proteolytic resolution of TOP2ccs (see Schellenberg et al. Science 2017 ref. #23). We have shown that the capacity of BPLF1 to stabilize TOP2ccs and rescue TOP2 toxicity is dependent on the DUB activity (i.e. it involves inhibition of TOP2 degradation by the proteasome) and requires TDP2 (i.e. it is dependent on hydrolysis of the TOP2-DNA adducts by TDP2). We have not directly addressed the role of TOP2 SUMOylation but, based on the literature and on the observation that BPLF1 does not alter the SUMOylation of TOP2, we propose that the persistent SUMOylation may play a role in granting to TDP2 access to the TOP2-DNA bond. We agree with the reviewer that, quite independently on the effect of BPLF1, the role of SUMOylation in the regulation of TOP2 is a very interesting topic and we have recently acquired a ZNF451 mutant that may help us address this issue. However, this analysis is not within the scope of the manuscript. Furthermore, we feel that the use of SUMOylation sites mutants would not significantly add to our current study of the mechanism of action of viral DUB and we have therefore not included the suggested experiment.
3. As stated in the text and figure legends the data of Dox- cells were pooled since in the absence of BPLF1, independently on whether the enzyme is catalytic active or inactive, the cells behave similarly, as also confirmed by the very small SD. We feel that this graphic presentation simplifies the figure and makes the message more direct. We have therefore not modified the presentation of the data in Fig. 4C and revised figure Fig. 6I and 7A.

**Minor issues**

1. We apologize for the inconsistency. The commas versus dots were corrected in all figures
2. We acknowledge the lack of clarity. Figure 3B was changed by moving the indication of treatment to the side of the graph.
3. Corrected as suggested
4. Corrected as suggested
5. The correct reference is now included in the supplementary table
6. Corrected as suggested

**Figure 1. Expression of γH2AX in untreated and etoposide treated TK6 and TK6^-/-^ cells**. Lack of TDP2 is associated with high endogenous levels of DNA damage. Representative micrographs from one representative experiment out of two are shown.

**TK6-TDP2^-/-^**

**TK6**

**control**

**Etoposide**

**γH2AX**

**γH2AX + DAPI**

**γH2AX**

**γH2AX + DAPI**

7. Corrected as suggested

**Figure 2. Catalytically active BPLF1 promotes EBV DNA replication and the release of infectious virus.** The productive virus cycle was induced in the indicated cell lines by treatment with Dox and viral DNA was measured by qPCR in cell pellets and DNAse treated culture supernatants harvested after 3 days. The mean ± SD of 2-3 independent experiments where the cell pairs were texted in parallel are shown.
